# Ultranarrow Photoluminescence from Individual Graphene Nanoribbons Showing Single-Photon Emission

**DOI:** 10.1021/acs.nanolett.6c00287

**Published:** 2026-03-27

**Authors:** Bernd K. Sturdza, Amit Pawbake, Clement Faugeras, Wenhui Niu, Ji Ma, Xinliang Feng, Moritz K. Riede, Lapo Bogani, Robert A. Taylor, Robin J. Nicholas

**Affiliations:** † Clarendon Laboratory, Department of Physics, 6396 University of Oxford, Parks Road, Oxford OX1 3PU, United Kingdom; ‡ Laboratoire National des Champs Magnetiques Intenses, 129851 CNRS-UJF-UPS-INSA, F-38042 Grenoble, France; § Center for Advancing Electronics Dresden (CFAED), Faculty of Chemistry and Food Chemistry, 9169 Technische Universität Dresden, Mommsenstrasse 4, 01062 Dresden, Germany; ∥ Max Planck Institute of Microstructure Physics, Weinberg 2, 06120 Halle, Germany; ⊥ Department of Materials, 6396 University of Oxford, 16 Parks Road, Oxford OX1 3PH, United Kingdom; # Departments of Chemistry and Physics, University of Florence, V. della Lastruccia, 50019 Sesto Fiorentino, Italy

**Keywords:** graphene nanoribbons, photoluminescence, single-photon emission, spectral diffusion, time-resolved PL, zero-phonon line, electron−phonon coupling

## Abstract

Graphene nanoribbons (GNRs) combine the remarkable optical and electronic properties of graphene with the presence of a tunable band gap, making them promising for optoelectronic applications. Here, we investigate the excitonic properties of individual cove-edge GNRs through microphotoluminescence (micro-PL) spectroscopy. We observe ultranarrow emission lines with full width at half-maximum as low as 24 μeV, demonstrating a reduction of inhomogeneous broadening by 3 orders of magnitude compared to GNR ensembles. Temperature-dependent PL reveals phonon-mediated broadening mechanisms, with electron–phonon coupling parameters in agreement with ensemble studies but with dramatically reduced line widths. Time-resolved PL suggests long-lived excitonic states, while spectral diffusion analysis demonstrates stable emission energies, highlighting the exceptional quality of these GNRs as single-photon emitters. The absence of intensity blinking and low Mandel parameters further support the robustness of the emission properties. Our findings establish cove-edge GNRs as promising candidates for quantum light sources and nanoscale optoelectronic applications.

## Graphical abstract

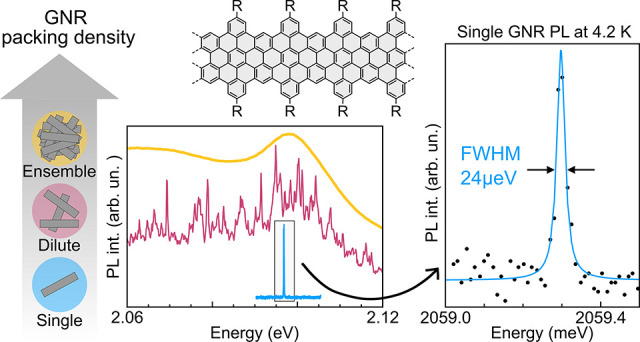

Carbon nanostructures have drawn a large amount of scientific attention due to their outstanding optical and electronic properties. However, the absence of a band gap in graphene and the presence of dark states in single-walled carbon nanotubes (CNTs) limits their potential for applications in optoelectronics, bioimaging, and solar energy conversion, as well as in quantum technologies for single-photon sources, upconversion, and sensing.^1–4^

Graphene nanoribbons (GNRs) combine the beneficial properties of graphene with an electronic band gap, which is necessary for many applications, such as light-emitting devices. In contrast to CNTs, GNRs possess edges to which side groups can be attached without disrupting their electronic structure to e.g. prevent aggregation. These edges can be controlled with atomic precision, allowing the tuning of optical and electronic properties.^5^ This enables GNRs to overcome the inherent PL quenching by dark states typically present in semiconducting carbon nanostructures. Dark excitons occur in carbon nanomaterials because of exchange- and symmetry-induced exciton fine-structure splitting associated with valley and spin degrees of freedom causing only one out of 16 possible exciton states to be bright.^6,7^

The edge modulation in cove-edge GNRs, which are GNRs with zigzag edges with an additional carbon hexagon present in every third spot on both sides (see Figure 1a), lifts the K-point degeneracy and the 3-fold edge symmetry causes zone-folding of the dark states into the Γ-point zone center, allowing all of the previously dark excitons to mix and become much more emissive.^8^

**Figure 1:**
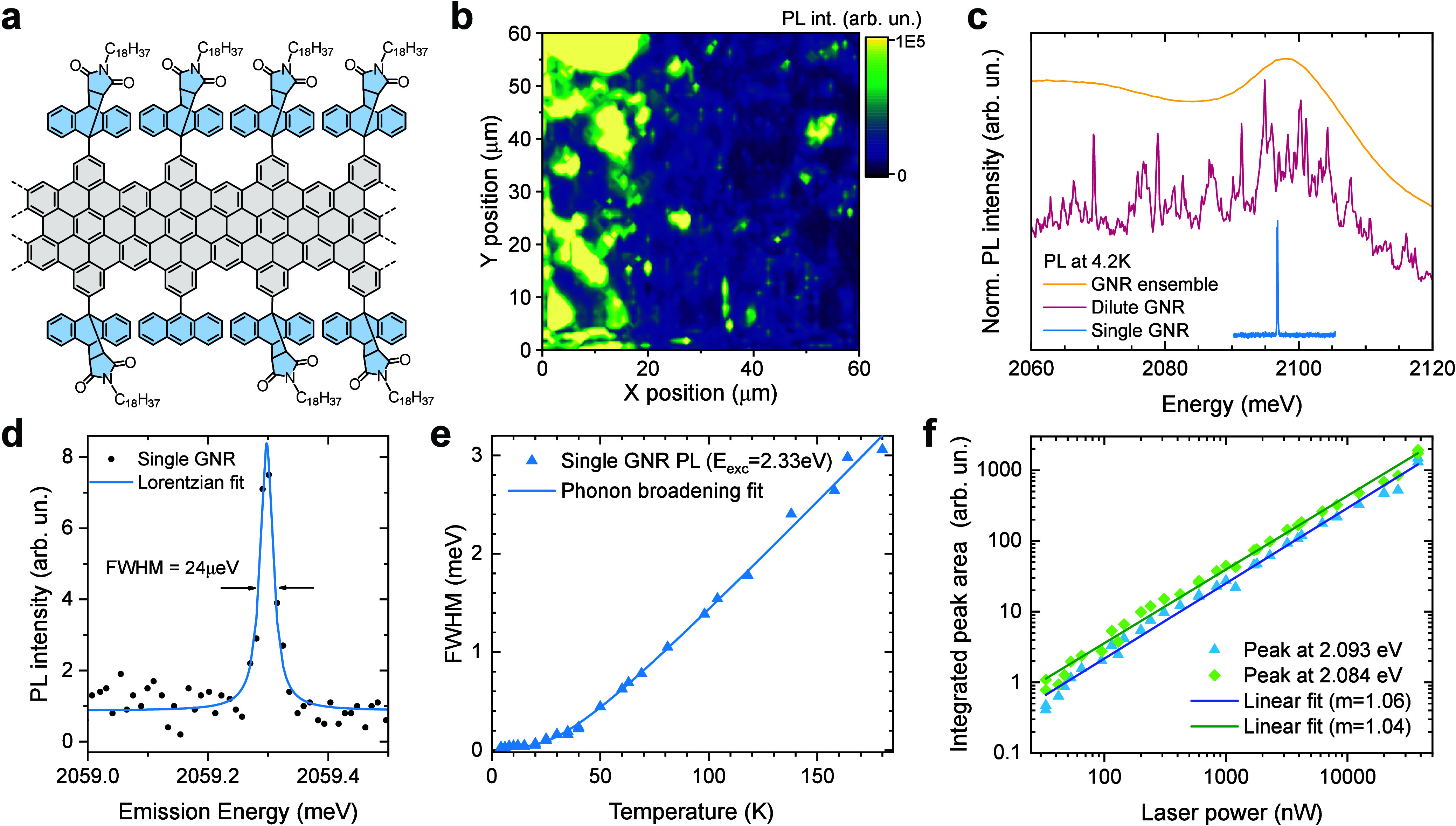
Photoluminescence from individual cove-edge graphene nanoribbons. (a) Molecular structure of the cove-edge GNR under study with *N-n*-octadecylmaleimide and anthracene side groups. (b) Micro-PL 2D map of a dilute GNR sample on SiO_2_ with 1 μm pixel size. The PL intensity was integrated in the ZPL-relevant spectral range (2.05–2.1 eV). (c) Normalized PL spectra of the same type of GNR at different concentrations. (d) High-resolution PL spectrum of a single GNR with Lorentzian fit to the data and resulting FWHM. (e) Temperature evolution of the single GNR ZPL line width under 2.25 eV excitation shown with fit line (see text). (f) Laser power dependence of the integrated PL peak area for two single GNRs.

Although the optical properties of GNRs with various edges, lengths, and widths have been extensively studied in theory, experimental data on the excitonic behavior of GNRs are largely missing.^9–11^ Existing work is generally limited to ensembles of GNRs which show a broadened optical response often dominated by defects^12^ or studies on surface-grown GNRs suffering from luminescence quenching and showing only STM-induced fluorescence.^7^

Here we report the photoluminescence of individual cove-edge GNRs. The cove-edge GNRs exhibit ultranarrow emission lines, packing density dependent electron–phonon coupling and strong indications of single-photon emission. We study the excitonic properties of individual GNRs at different temperatures, excitation energies and intensities revealing graphitic (G) mode and radial-breathing-like modes (RBLM) as the dominant phonon modes and homogeneous broadening with an electron–phonon coupling strength and phonon energy consistent with previous results as the main broadening mechanism. The inhomogeneous broadening is dramatically reduced compared to ensemble measurements. Time resolved PL experiments show a slow and a fast component with exciton lifetimes decreasing with increasing GNR packing density on the substrate. In contrast to previous studies, the GNRs can be deposited on any substrate by simple drop casting at sufficient dilution.

To study the excitonic properties of individual GNRs, we chose a micro-PL setup with a 1 μm focal spot and soluble cove-edge GNRs,^13^ see Figure 1a. The GNRs are synthesized via Diels–Alder cycloaddition and carry bulky *N-n*-octadecylmaleimide side groups with a grafting ratio of 77%. The remaining sites are occupied by anthracene side groups. The high solubility of these samples allows us to precisely dilute them to very low densities in chloroform. We find that at concentrations of 10^–4^ mg/mL we can dropcast 5 μL onto a SiO_2_ wafer and create a sparse network of GNRs that can be individually resolved in PL spectra of diffraction-limited spots. A far-field 2D PL map of such a wafer is shown in Figure 1b. The energy-integrated 60 × 60 μm micro-PL map with a pixel size of 1 × 1 μm possesses areas of higher GNR packing density with a bright PL response and areas of low GNR packing density with weaker PL from one or two individual spots. We compare the micro-PL spectra of high and low GNR packing density areas in Figure 1c to a GNR ensemble spectrum taken at a much higher concentration (10^–2^ mg/mL) similar to the ensemble data we have presented previously.^8^ The GNR ensemble PL data shows a single smooth and broadened zero-phonon line (ZPL) peak with a full width at half-maximum (FWHM) of about 17 meV. In contrast, the ZPL of individual GNRs can be resolved in the micro-PL data of the dilute sample. In the higher packing density areas, tens of GNRs can be resolved from their individual ZPL positions. The small shifts in ZPL peak position are likely caused by differences in the microscopic environment of the GNRs, see further discussion below. In the lower packing density areas, the micro-PL spectrum of an individual GNR can be detected. These spectra consist of a single, extremely narrow ZPL peak with detected FWHM values as low as 24 μeV (Figure 1d). Note that these line widths are still resolution-limited with a 1500 + 1500 + 2000 l/mm triple grating 0.75 m spectrometer in additive mode and are probably even narrower. This line width is almost 3 orders of magnitude smaller than for the GNR ensemble and still more than 20 times narrower than the narrowest line widths of single GNRs reported to date, which were measured in STM-induced electroluminescence experiments.^7,14^ The observed large difference between line widths for ensembles and individual chromophores is similar to carbon nanotubes, where inhomogeneous broadening also strongly affects ensemble samples.^15^

Temperature-dependent micro-PL measurements of the ZPL line width (Figure 1e) provide insights into the internal processes involved in the emission. The *T*-dependence of the line-broadening parameter Γ­(*T*) can be fitted with the expression Γ (*T*) = Γ_0_ + Γ_ph_ = Γ_0_ + γ (*e* ^*E*_ph_/*k*_B_ *T*^–1) ^–1^ and yields the inhomogeneous broadening linked to scattering on terminations,^16^ Γ_0_ = 0.033 ± 0.030 meV, the homogeneous broadening produced by phonon scattering^17^ Γ_ph_, and an electron–phonon coupling strength γ = 2.0 ± 0.4 meV with phonons at energy *E*_ph_ = 4.8 ± 0.7 meV. Interestingly, the coupling strength and phonon energy are nearly the same as we found for ensemble measurements on the same GNRs,^8^ but the inhomogeneous broadening is reduced by a factor of 500. This vast difference between the inhomogeneous line width of a single ribbon and GNR ensembles suggests that the mechanism for photon emission in GNRs is connected to long-lived excitons in a low energy state. This further suggests that any individual GNR has sufficiently long exciton lifetimes and diffusion lengths to emit from probably only a single emission site governed by the local energy landscape of each individual GNR, in equivalence to observations on individual carbon nanotubes.^18^ The large relative error in the inhomogeneous broadening parameter Γ_0_ obtained from the fit indicates once more that the actual line widths of the ZPL are likely narrower than our resolution-limited results.

The excitation power dependence of the integrated PL peak area exhibits a linear relationship with slope 1 over the entire studied range, see Figure 1f. This indicates the absence of saturation and a low defect density in our samples. A rough estimate for the PL quantum efficiency suggests that the emission rates are consistent with a value approximately 2–10 times higher than the measured room temperature values for ensemble measurements at 300 K of 7%.^8^

Many single-photon emitters such as quantum dots suffer from two effects, namely spectral diffusion and intensity blinking. These effects can be observed by taking a time series of consecutive PL spectra. We have measured a series of 500 PL spectra with 250 ms acquisition time each for a single GNR. The ribbon shows a single narrow emission line at 2093 meV (Figure 2a). Upon increasing the energy resolution, we find that the spectral diffusion of the PL energy is within about 0.8 meV, see Figure 2b, a relative change in emission energy of about 0.04%.

**Figure 2:**
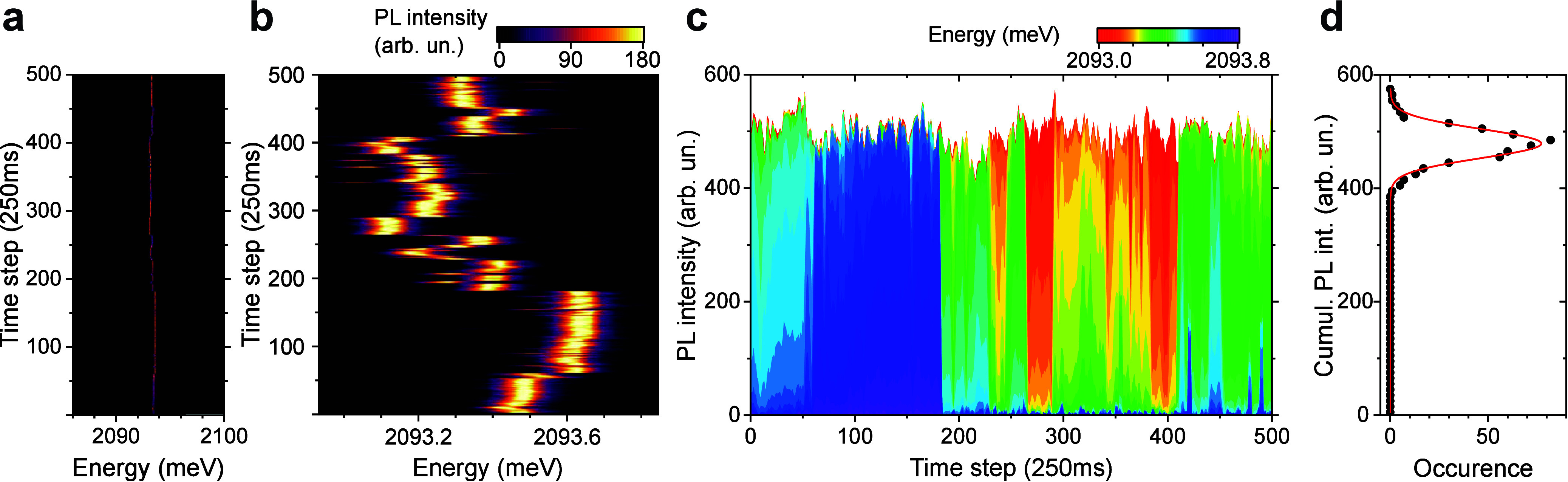
Spectral diffusion of single GNR PL emission at 4 K. (a, b) Time series of PL emission for a single GNR in 250 ms steps at different energy resolutions. The sample is illuminated with 5 μW of 2.089 eV radiation and the scale shows counts per pixel for the acquisition time (250 ms). (c) Temporal evolution of the PL intensity distribution over the energy range shown in b). (d) Histogram of the cumulative PL intensity as shown in (c) (bin size = 5 arb. un.) with Gaussian fit.

Spectral diffusion is a common effect in nanocrystals and typically caused by Stark effects, i.e. charging of the sample or substrate.^19–22^ While the hopping of the emission energy within the observed range appears to be random, there are discrete energies around which the emission fluctuates. These discrete energies are separated by about 0.1–0.2 meV and the most prominent one is around 2093.65 meV. The temporal evolution of the PL intensity is given in Figure 2c, where each individual pixel on the energy axis (pixel width 35 μeV) in Figure 2b is assigned a separate color and the intensities are stacked. This clearly demonstrates that the GNR only emits at one energy at a given time, a quality typically found in single-photon emitters.

The stability of the PL intensity over time can be quantified with the Mandel parameter $Q=(\frac{\left\langle n^{2}\right\rangle -\left\langle n^{2}\right\rangle }{\left\langle n\right\rangle })-1$ where *n* is the number of photons observed in a given time period (here 250 ms) and ⟨*n*⟩ is the mean value of *n* over the whole series. The value of *Q* provides a measure of the deviation of the PL intensity distribution from the Poissonian distribution of detector shot noise. Values close to *Q* = 1 show that the system is in the shot noise limit.^23^ We find *Q* = 1.6, suggesting the single GNR PL emission is basically free from long time scale fluctuations and is shot noise limited. A histogram of the PL intensity (bin size = 5) is shown with a Gaussian fit in Figure 2d, the fit returns a line width of FWHM = 60 ± 1 arb. un. which is about 13% of the mean cumulative PL intensity.

The low *Q* value suggests that the PL emission of individual GNRs originates from a two state system. The presence of photoluminescence bleaching within hours and relatively low PL intensities prevented successful observation of photon antibunching for our samples. However, a previous study on individual color centers in doped SWCNTs found that values of *Q* < 5 correlate with single-photon emission, whereas no single-photon emission was reported for SWCNTs with higher *Q* values which can be up to several hundreds.^24,25^ It is worth noting, that our GNR samples achieve these extremely low Q values in a pristine state without the presence of color centers. This highlights the purity of our samples and underlines their potential as promising emitter material.

To study the internal processes leading to the recombination of excitons, we perform photoluminescence excitation (PLE) spectroscopy and time-resolved (TR) PL. By comparing a large number of single GNR PLE data, we find that the position of the ZPL PL peak is generally not affected by the excitation energy, see Figure 3b. The PL intensity, however, is strongly affected by the excitation energy (Figure 3a) showing three peaks roughly 1G, 1G+1RBLM, and 1G+2RBLM phonon energies above the ZPL emission energy. All three peaks show additional shoulders on the higher energy side, suggesting that phonon scattering with an additional low energy mode is present. The PLE suggests that phonon-assisted band edge to band edge transitions are the dominant recombination process, which matches observations on ensembles of the same type of GNR.^8^

**Figure 3:**
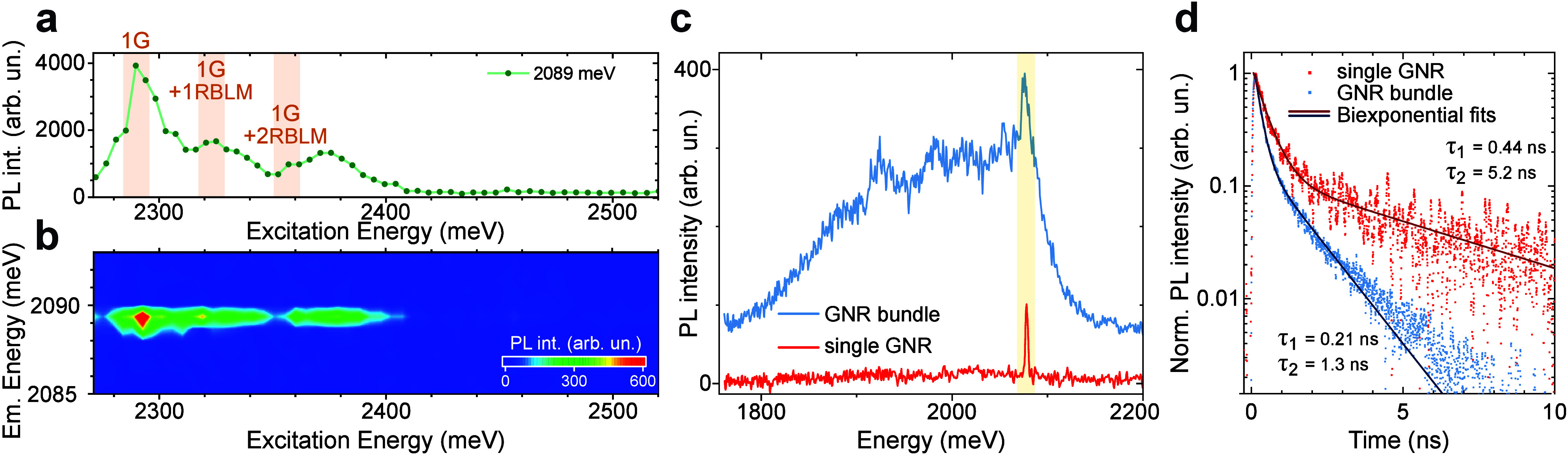
PL excitation and time-resolved PL spectroscopy on single GNRs at 4 K. (a) Integrated PL intensity of a single GNR for different excitation energies. The three peaks are 1G and 1G+RBLM phonon energies above the ZPL emission energy. (b) False color PLE map of the single GNR shown in a) above. (c) PL spectra of a single GNR and GNR bundle. The yellow area highlights the ZPL peak which was used for TRPL experiments. (d) Time-resolved PL spectra of a single GNR and GNR bundle fitted with biexponential decays, the resulting lifetimes are given.

The line width of the PLE peaks in direction of excitation energy remains relatively broad (30 meV) compared to the line width of the emission but is still about 5 times narrower than for ensemble samples (150 meV).^8^ This behavior again resembles carbon nanotubes where similar FWHM values have been reported for PLE absorption of individual polymer-wrapped SWCNTs.^18^

For the purpose of this study, we examined a large number of individual GNRs. Interestingly, we found that the cw PL spectrum appears quite sensitive to the packing density of GNRs. While a single, isolated GNR only produces a single emission line corresponding to the zero-phonon line, a bundle of GNRs already produces a phonon wing in the PL, see Figure 3c. Although the ZPL emission remains at the same energy position, its intensity is enhanced and additional emission at lower energies arises for the GNR bundle, likely caused by the larger number of available excitonic and vibronic states.

Time-resolved PL measurements of the same single GNR and bundle show that the lifetimes are significantly longer in the single ribbon (Figure 3d). The decays of both samples follow a biexponential behavior with a fast component dominating the first few ns and a slower decay of 1.3 ns for the bundle and 5.2 ns for the single GNR. As we have previously reported, the fast component is associated with Franck–Condon type electron–phonon coupling.^8^ The slow component of several ns is comparable to the lifetimes observed in suspended carbon nanotube quantum dots.^26^ The faster decay of GNR bundles can again be assigned to the larger number of available states and possibly tunneling.

Calculating the lifetime broadening for the fast decay of a single GNR τ_1_ = 0.44 ns yields an FWHM of *ΔE* = ℏ/ τ = 1.5 μeV suggesting that the observed ZPL line width of 24 μeV is still not lifetime limited but limited by the spectrometer resolution or nonradiative dephasing mechanisms, e.g. phonons, charge noise, or spectral diffusion.

Upon studying the PL emission of a large number of individual GNRs, we noticed an energy dispersion in their zero-phonon line emission. This can be visualized by replotting the spatial map of Figure 1b with an emission energy resolution, see Figure 4a. We find ribbons with sharp emission lines emitting anywhere in the range of 2063–2105 meV. Emission at lower energies is caused by phonon-assisted transitions as we have previously shown^8^ and are thus excluded in this figure.

**Figure 4:**
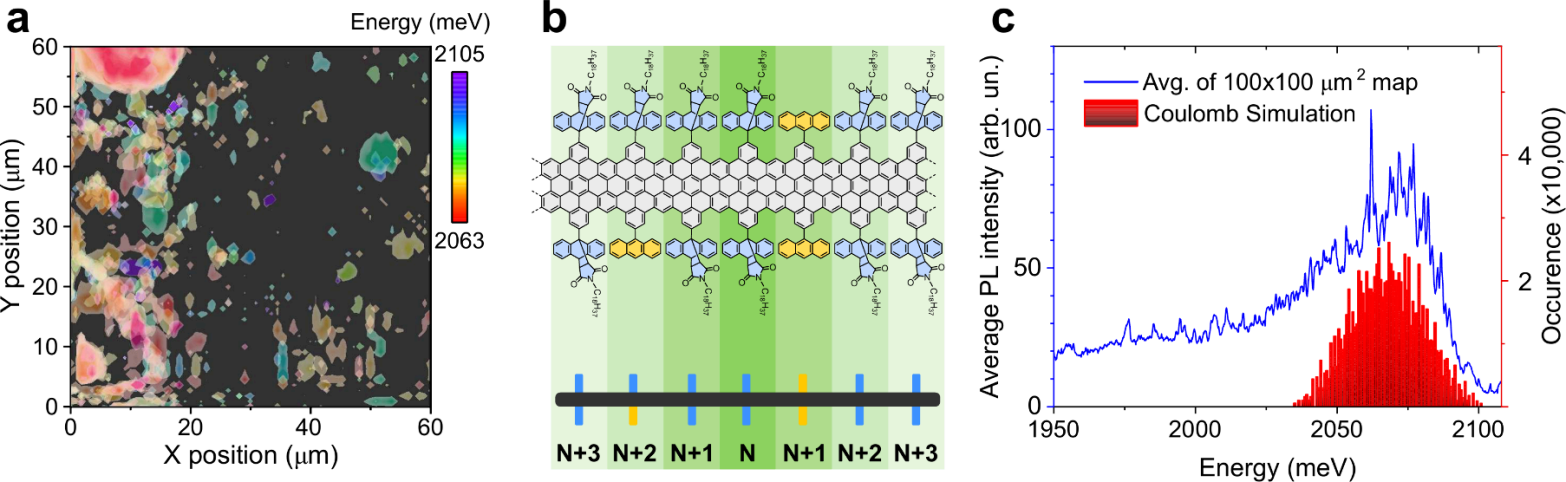
GNR zero-phonon line emission energy dispersion. (a) Energy-resolved false color plot of the same spatial PL map shown in Figure 1b. (b) Schematic illustrating the Coulomb-type simulations which consider the effect of the 14 nearest side groups on the local band gap. (c) Average of 10,000 PL spectra taken in the spatial PL map shown in (a) shown together with the distribution of emission energies produced by the simulation.

Small differences in the emission energy of quantum emitters are typically caused by inhomogeneities in the local environment. The band gap of GNRs is to a first order determined by their lateral confinement, i.e. their width and edge structure. Since the range of ZPL emission lines only spans about ± 20 meV which is about ± 1% of the band gap, we conclude that this is caused by a second order effect rather than differences in the lateral confinement.

A similar argument has previously been made for excitons in suspended carbon nanotubes where exciton localization is thought to improve the quantum yield.^26^ GNR length-related effects can be ruled out as reason for the ZPL energy dispersion, since the exciton confinement length for our GNRs is on the order of 1–2 nm which is much smaller than the GNR lengths of 10–371 nm.^13^ This is consistent with computational results suggesting that the nanoribbon length has a negligible effect on the band gap energy at lengths beyond 5–10 nm.^27–29^

Secondary effects potentially affecting the band gap include, as previously mentioned, the local environment of the GNRs which is determined by the orientation on the substrate and the side groups. The two types of side groups attached to the GNRs, anthracene (A) and *N-n*-octadecylmaleimide (B), are randomly allocated with a probability of 23% and 77%, respectively. The two groups differ substantially in size and weight and are therefore expected to have an effect on the local energy landscape of the GNR.

To support this argument, we perform a simple simulation. We assume the excitons in our GNRs are delocalized over several nanometers, in accordance with excitons in CNTs,^30,31^ and their emission energy affected by the GNR side groups in close proximity. For the sake of simplicity, for an exciton positioned at a given site *N* we only consider the nearest side groups up to site *N* ± 3, see Figure 4b. We then randomly occupy the sites with the two side groups A and B, according to their grafting ratio and further assume that sites with group A reduce the band gap, whereas sites with group B have no effect. Finally, we assume the strength of the effect follows a Coulomb type behavior and decays quadratically with the distance to site *N*. Now, we simulate 1 million GNRs accordingly and show the resulting distribution of emission energies together with the average of 10,000 PL spectra taken from a spatial map, see Figure 4c. We find that the dispersion of emission energies is reproduced well by our simple model. Emission energies below 2.06 eV are part of the phonon side wing and thus not considered here.

In this study, we have demonstrated the exceptional photoluminescence properties of individual cove-edge graphene nanoribbons. The observation of ultranarrow emission lines, which are 3 orders of magnitude smaller than previously observed in PL studies of graphene nanoribbons, demonstrates that individual GNRs are free from the inhomogeneous broadening that has previously obscured their excitonic behavior and show the characteristics of quantum dots. Temperature-dependent and time-resolved PL measurements show that the emission is dominated by phonon-mediated broadening, with coupling strengths consistent with ensemble studies at high temperatures but dramatically reduced line widths as the temperature falls. We further observe highly stable emission energies with repeatable spectral diffusion between states only a few tens or hundreds of μeV apart but no intensity blinking, indicating robust excitonic emission from a two-level system. The low Mandel parameters we measure provide strong evidence for photon statistics corresponding to a two-state system in the single-photon emission regime. The most likely origin of the spectral diffusion is due to Stark shifts associated with adjacent charging sites suggesting that it may be possible to construct a gated structure which can control the emission energies for potential quantum computing applications. The narrow quantum dot-like emission lines occur over a range of approximately 200 meV in an ensemble of ribbons suggesting that hundreds of distinguishable states might be addressable on a single chip. Our findings suggest that cove-edge GNRs represent a new class of high-quality, carbon-based quantum emitters. Further engineering of the edge structure and chemical functionalization of GNRs will allow tailoring of their optical properties for specific applications, such as integrated photonic circuits and single-photon sources for quantum communication.

## Sample Preparation

Cove-edge GNRs decorated with the Diels–Alder cycloadduct of an anthracenyl unit and *N-n*-octadecylmaleimide (GNR-AOM) were prepared according to reported procedures.^8,13^ Ribbons have a length distribution of *L* = 10–371 nm.

To prepare single GNRs on a substrate, the GNR powder is dissolved in chloroform at a concentration of 0.1 mg/mL. After 10 min, the solution is filtered through a 0.2 μm syringe filter to remove impurities. Next, the solution is diluted down to a concentration of 10^–4^ mg/mL and now ready to be deposited. The Si/SiO_2_ substrate is cleaned in a sonication bath in acetone and in the second step in chloroform. The substrate is blown dry with a nitrogen gun after removal from the chloroform bath. Next, 5 μL of the diluted nanoribbon solution is deposited with a small pipet on to the surface of the substrate. Immediately after this, the droplet is blown across the substrate with the nitrogen gun until the solvent is fully evaporated. This is to prevent the coffee ring effect and ensure individual GNRs do not bundle up during solvent evaporation.

## Micro-PL

Low-temperature microphotoluminescence measurements were taken with the sample immersed in helium gas at 4.2 K. Continuous wave excitation was produced by an NKT Photonics supercontinuum laser tunable from 400 to 1000 nm directed through a monomode fiber with 5 μm diameter core, in the range 400–570 nm. For experiments other than the PLE measurements, the excitation wavelength was set to coincide with the lowest energy peak in the PLE (1G phonon above the ZPL) which corresponds to 540–552 nm. After passing a beam splitter the excitation was focused on the sample surface with a spot size of 1 μm. The sample was manipulated with submicron precision with an Attocube *xyz*-piezostage. The piezostage was used to scan the position of the excitation (step sizes 0.5 – 1 μm), and a spectrum was taken for each pixel to create a detailed map of the GNRs on the sample. These hyperspectral images contain a great deal of information including the size, position, bundling, and specific energy levels of the GNRs.

Emission was collected using free beam optics from the sample to the spectrometer and detected by a Princeton Instruments Acton Trivista 0.75 m spectrometer with a 500 l/mm grating used for the PLE spectra and a 1500 + 1500 + 2000 l/mm triple grating in additive mode for the high-resolution spectra.

## Time-Resolved Micro-PL

Time resolved photoluminescence measurements were acquired with a Picoquant PMA-40 PMT TCPC detector with a response time of 105 ps. The TCPC card was a Picoquant Picoharp 400 with 4 ps bin time. The spectrograph was an Andor Shamrock 500i 0.5 m system with an IDus CCD, or the PMA TCPC detector on dual ports. The grating was either a 1200 l/mm one for the PL spectra or 300 l/mm for the time-resolved measurements. The excitation laser was a 10 ps pulse width frequency doubled YAG laser operating at 532 nm with a rep rate of 40 MHz, or sometimes we used a CW 532 nm YAG laser for the spectral measurements. When we did temperature dependent line width measurements the cryostat used was an Attodry 800 with a base temperature of 6 K.

## References

[1] Martel R., Schmidt T., Shea H. R., Hertel T., Avouris P. (1998). Single- and
multi-wall carbon nanotube field-effect transistors. Appl. Phys. Lett..

[2] Arnold M. S., Blackburn J. L., Crochet J. J., Doorn S. K., Duque J. G., Mohite A., Telg H. (2013). Recent developments in the photophysics
of single-walled carbon nanotubes for their use as active and passive
material elements in thin film photovoltaics. Physical Chemistry Chemical Physics.

[3] Ferrari A. C. (2015). Science and technology
roadmap for graphene, related two-dimensional
crystals, and hybrid systems. Nanoscale.

[4] Que M., Zhang B., Chen J., Yin X., Yun S. (2021). Carbon-based
electrodes for perovskite solar cells. Materials
Advances.

[5] Narita A. (2014). Synthesis of structurally
well-defined and liquid-phase-processable
graphene nanoribbons. Nat. Chem..

[6] Ando T. (2006). Effects of
valley mixing and exchange on excitons in carbon nanotubes with Aharonov-Bohm
flux. J. Phys. Soc. Jpn..

[7] Jiang S., Neuman T., Boeglin A., Scheurer F., Schull G. (2023). Topologically
localized excitons in single graphene nanoribbons. Science.

[8] Sturdza B. K., Kong F., Yao X., Niu W., Ma J., Feng X., Riede M. K., Bogani L., Nicholas R. J. (2024). Emissive
Brightening in Molecular Graphene Nanoribbons by Twilight States. Nat. Commun..

[9] Yang L., Cohen M. L., Louie S. G. (2007). Excitonic
Effects in the Optical
Spectra of Graphene Nanoribbons. Nano Lett..

[10] Prezzi D., Varsano D., Ruini A., Marini A., Molinari E. (2008). Optical properties
of graphene nanoribbons: The role of many-body effects. Phys. Rev. B.

[11] Deilmann T., Rohlfing M. (2017). Huge Trionic Effects in Graphene Nanoribbons. Nano Lett..

[12] Ma C., Xiao Z., Puretzky A. A., Wang H., Mohsin A., Huang J., Liang L., Luo Y., Lawrie B. J., Gu G., Lu W., Hong K., Bernholc J., Li A.-P. (2020). Engineering
Edge States of Graphene Nanoribbons for Narrow-Band Photoluminescence. ACS Nano.

[13] Niu W. (2023). Exceptionally clean single-electron transistors from solutions of
molecular graphene nanoribbons. Nature materials.

[14] Chong M. C., Afshar-Imani N., Scheurer F., Cardoso C., Ferretti A., Prezzi D., Schull G. (2018). Bright Electroluminescence from Single
Graphene Nanoribbon Junctions. Nano Lett..

[15] Htoon H., O’Connell M. J., Cox P. J., Doorn S. K., Klimov V. I. (2004). Low temperature
emission spectra of individual single-walled carbon nanotubes: Multiplicity
of subspecies within single-species nanotube ensembles. Phys. Rev. Lett..

[16] Rudin S., Reinecke T. L., Segall B. (1990). Temperature-dependent
exciton linewidths
in semiconductors. Phys. Rev. B.

[17] Viswanath A. K., Lee J. I., Kim D., Lee C. R., Leem J. Y. (1998). Exciton-phonon
interactions, exciton binding energy, and their importance in the
realization of room-temperature semiconductor lasers based on GaN. Physical Review B - Condensed Matter and Materials Physics.

[18] Alexander-Webber J. A., Faugeras C., Kossacki P., Potemski M., Wang X., Kim H. D., Stranks S. D., Taylor R. A., Nicholas R. J. (2014). Hyperspectral
imaging of exciton photoluminescence in individual carbon nanotubes
controlled by high magnetic fields. Nano Lett..

[19] Empedocles S. A., Bawendi M. G. (1997). Quantum-confined
stark effect in single cdSe nanocrystallite
quantum dots. Science.

[20] Empedocles S. A., Bawendi M. G. (1999). Influence of spectral
diffusion on the line shapes
of single CdSe nanocrystallite quantum dots. J. Phys. Chem. B.

[21] Braam D., Mölleken A., Prinz G. M., Notthoff C., Geller M., Lorke A. (2013). Role of the ligand layer for photoluminescence spectral diffusion
of CdSe/ZnS nanoparticles. Physical Review B
- Condensed Matter and Materials Physics.

[22] Ihara T., Kanemitsu Y. (2014). Spectral diffusion
of neutral and charged exciton transitions
in single CdSe/ZnS nanocrystals due to quantum-confined Stark effect. Physical Review B - Condensed Matter and Materials Physics.

[23] Margolin G., Protasenko V., Kuno M., Barkai E. (2006). Photon counting statistics
for blinking CdSe-ZnS quantum dots: A lévy walk process. J. Phys. Chem. B.

[24] Ma X., Hartmann N. F., Baldwin J. K., Doorn S. K., Htoon H. (2015). Room-temperature
single-photon generation from solitary dopants of carbon nanotubes. Nat. Nanotechnol..

[25] Orcin-Chaix L., Campidelli S., Rondin L., Fossard F., Bretenaker F., Chassagneux Y., Voisin C., Lauret J. S. (2020). Photostability of
Single-Walled Carbon Nanotubes/Polymer Core-Shell Hybrids as Telecom
Wavelength Emitters. ACS Applied Nano Materials.

[26] Hofmann M. S., Glückert J. T., Noé J., Bourjau C., Dehmel R., Högele A. (2013). Bright, long-lived
and coherent excitons in carbon
nanotube quantum dots. Nat. Nanotechnol..

[27] Kimouche A., Ervasti M. M., Drost R., Halonen S., Harju A., Joensuu P. M., Sainio J., Liljeroth P. (2015). Ultra-narrow
metallic armchair graphene nanoribbons. Nat.
Commun..

[28] Zdetsis A. D., Economou E. (2017). Rationalizing and reconciling
energy gaps and quantum
confinement in narrow atomically precise armchair graphene nanoribbons. Carbon.

[29] Talirz L., Söde H., Kawai S., Ruffieux P., Meyer E., Feng X., Müllen K., Fasel R., Pignedoli C. A., Passerone D. (2019). Band Gap of
Atomically Precise Graphene Nanoribbons
as a Function of Ribbon Length and Termination. ChemPhysChem.

[30] Lüer L., Hoseinkhani S., Polli D., Crochet J., Hertel T., Lanzani G. (2009). Size and mobility of excitons in (6, 5) carbonnanotubes. Nat. Phys..

[31] Mann C., Hertel T. (2016). 13 nm Exciton Size in (6,5) Single-Wall Carbon Nanotubes. J. Phys. Chem. Lett..

